# A truncating *ELF4* variant in a pediatric patient with autoinflammatory disease and cell type-specific immune alterations

**DOI:** 10.3389/fimmu.2026.1859084

**Published:** 2026-09-07

**Authors:** Qianlu Zhang, Yatang Lei, Lina Zhou, Zhiyong Zhang, Xuemei Tang, Yunfei An, Xiaodong Zhao, Hongqiang Du

**Affiliations:** 1 Department of Rheumatology & Immunology Children’s Hospital of Chongqing Medical University, National Clinical Research Center for Children and Adolescents’ Health and Diseases, Ministry of Education Key Laboratory of Child Development and Disorders, Chongqing, China; 2 Chongqing Key Laboratory of Child Rare Diseases in Infection and Immunity, Chongqing, China; 3 Jiangxi Children’s Medical Center, Jiangxi Hospital Affiliated to Children’s Hospital of Chongqing Medical University, Jiangxi Maternal and Child Health Hospital, Nanchang, Jiangxi, China

**Keywords:** autoinflammatory diseases, ELF4, inborn errors of immunity, loss-of-function variant, single-cell RNA sequencing

## Abstract

**Introduction:**

ELF4 is an ETS family transcription factor involved in immune regulation, including antiviral responses and inflammatory signaling. Germline loss-of-function variants in ELF4 cause deficiency in ELF4, X-linked (DEX), a disorder characterized by recurrent mucocutaneous inflammation and variable immune abnormalities. To date, ELF4-related disease has been reported only in association with germline mutations. Here, we report a pediatric patient with autoinflammatory disease harboring a truncating *ELF4* variant (c.239T>A, p.L80*).

**Methods:**

Clinical and genetic evaluations were performed in the patient and his family. The *ELF4* c.239T>A variant was assessed by Sanger sequencing in peripheral blood and buccal mucosa samples. ELF4 protein expression was examined in patient-derived cells and ectopic expression of wild-type and mutant ELF4 constructs in HEK293T cells by immunoblotting. Full-length single-cell RNA sequencing was performed on whole-blood leukocytes to compare mutant-assigned and wild-type-assigned immune cells within the same individual.

**Results:**

An *ELF4* truncating variant (c.239T>A, p.L80*) was detected in peripheral blood but was not detected in parental blood samples or the patient’s buccal mucosa by Sanger sequencing. Given the limited sensitivity of Sanger sequencing, the developmental origin and tissue distribution of the variant could not be definitively determined. A reduced full-length ELF4 signal was observed in patient-derived PBMCs and in the p.L80* HEK293T overexpression system, together with impaired transcriptional activity in an IFN-β promoter reporter assay. Single-cell transcriptomic analysis identified cells with p.L80*-supporting transcripts across multiple immune lineages and suggested cell type-specific transcriptional alterations. Pathway analyses indicated reduced antiviral and interferon-related programs in mutant-assigned NK cells. Exploratory analysis of mutant-assigned CD16^+^ monocytes also suggested inflammation-related pathway alterations, although the limited number of allele-informative cells warrants cautious interpretation.

**Conclusions:**

ELF4 is an ETS family transcription factor that plays an important role in immune regulation. Our study identifies a functionally impaired *ELF4* p.L80* variant in a pediatric patient with autoinflammatory disease and provides preliminary evidence of cell type-specific transcriptional alterations associated with this variant.

## Introduction

ELF4 is a member of the ETS family of transcription factors (1) and exerts cell type-specific functions across innate and adaptive immune compartments. ELF4 promotes type I interferon induction and antiviral defense (1). In NK cells, it is required for normal development, terminal maturation, and cytotoxic function (2, 3). In macrophages, ELF4 sustains anti-inflammatory transcriptional programs while limiting the induction of inflammatory amplifiers (3). In T cells, ELF4 regulates naïve CD8^+^ T-cell quiescence (4) and trafficking and restrains the differentiation of CD4^+^ T cells toward the Th17 lineage (3, 5). These established cell type-specific functions provide a biological framework for understanding the heterogeneous immune abnormalities associated with ELF4 deficiency. Germline loss-of-function mutations in *ELF4* have been identified as the genetic cause of deficiency in ELF4, X-linked (DEX) (3, 6–13), a disorder characterized by recurrent mucocutaneous inflammation, systemic inflammatory manifestations, and variable immune abnormalities. Clinical presentations of DEX are heterogeneous, ranging from isolated mucosal ulcers to severe inflammatory bowel disease-like phenotypes, highlighting the context-dependent role of ELF4 in immune homeostasis. To date, ELF4-related disease has been exclusively associated with germline mutations. Given the detection of the variant in peripheral blood but not in parental blood samples or the patient’s buccal mucosa, a possible somatic mosaic origin was considered, although this could not be definitively established with the available samples.

Somatic mutations have emerged as important contributors to immune-mediated diseases (14–19). Single-cell transcriptomic approaches have further enabled cell type-specific dissection of monogenic and mosaic autoinflammatory disorders (20–23). Comparing mutation-associated transcriptional states within the same individual provides a particularly informative framework for resolving cell type-specific effects while minimizing interindividual variability (20, 23). In this context, full-length single-cell RNA sequencing has become a valuable approach for resolving cell type–specific transcriptional alterations associated with somatic mutations (24). Here, we report a pediatric patient harboring a truncating *ELF4* p.L80* variant and combine genetic, functional, and single-cell transcriptomic analyses to characterize its associated immune alterations.

## Materials and methods

### Patient

The Ethics Committee of Children’s Hospital of Chongqing Medical University approved the study. Written informed consent for participation in the study was obtained from the patient’s parents. Blood and buccal mucosa samples from the patient and family members were collected for genetic studies. Fresh whole blood from the patient was additionally obtained for single-cell RNA sequencing. All procedures were performed in accordance with the Declaration of Helsinki.

### Genetic analyses

Genomic DNA was isolated from peripheral blood samples and buccal mucosa using the QIAamp DNA Mini Kit (Qiagen GmbH, Germany) according to the manufacturer’s instructions. Disease-causing mutations were screened using whole-exome sequencing (WES) (Fujun Genetics, Inc.). The candidate *ELF4* variant was confirmed by Sanger sequencing using the following primers: seq F:5’- CATAGCTGGAAGACCCCTC-3’, seq R:5’-CCCCTCCTTACCTAACTCTCC-3’.

### Construction of plasmids

The 7.1-pCMV-3×Flag expression vector encoding wild-type human ELF4 was generated as previously described (6). Point mutations were introduced into the wild-type constructs by overlap-extension PCR, and the resulting plasmids were confirmed by Sanger sequencing.

### Cells

HEK293T cells (ATCC, CRL-11268) were cultured in Dulbecco’s modified Eagle medium (DMEM) supplemented with 10% fetal bovine serum at 37 °C in a humidified incubator with 5% CO2.

### Luciferase reporter assay

As previously described (6, 7), HEK293T cells were seeded in 24-well plates and transiently transfected with 50 ng of the luciferase reporter plasmid together with equal total amounts of the indicated expression plasmids or empty-vector control. As an internal control, 10 ng of pRL-TK plasmid was co-transfected. After 24 h, luciferase activity was measured using the Dual-Luciferase Reporter Assay System (Promega) with a TD-20/20 luminometer (Turner Designs), according to the manufacturers’ instructions. Firefly luciferase activity was normalized to Renilla luciferase activity.

### PBMC isolation and immunoblot analysis

Peripheral blood mononuclear cells (PBMCs) were isolated from whole blood by density-gradient centrifugation using Ficoll-Paque Premium (GE Healthcare, Sweden). Whole-cell lysates including PBMCs and HEK293T were prepared in 1× RIPA buffer supplemented with protease inhibitors (Beyotime). After clarification by centrifugation, the lysates were mixed with 5× SDS loading buffer and denatured at 100 °C for 10 min. The samples were separated by 10% SDS-PAGE and transferred onto PVDF membranes. The membranes were incubated with primary antibodies against ELF4 (Abcam, ab96075), FLAG (Selleck, F0973), GAPDH, or α-tubulin, followed by HRP-conjugated secondary antibodies (Proteintech). Protein bands were visualized using enhanced chemiluminescence.

### Full-length single-cell RNA sequencing

Full-length single-cell RNA sequencing (25, 26) [SeekOne ^®^ DD scFAST-seq (27)] was performed on leukocytes prepared from fresh whole-blood samples obtained from the patient harboring the *ELF4* p.L80* variant. Single-cell RNA-seq libraries were constructed using the SeekOne^®^ Single Cell Whole Transcriptome Kit (SeekGene) following the manufacturer’s instructions, loaded onto the SeekOne^®^ DD Chip S3, and sequenced on an Illumina NovaSeq 6000 platform with paired-end 150 bp reads.

### Preprocessing, quality control, and cell clustering

Raw sequencing data were processed using fastp to remove adapter sequences and low-quality reads, followed by analysis with the SeekSoul pipeline for cell barcode and unique molecular identifier (UMI) demultiplexing and alignment to the human reference genome (GRCh38). Gene expression matrices were generated based on UMI counts. Cells with low UMI counts or excessive mitochondrial gene expression were excluded to ensure data quality. Downstream analyses were performed using the Seurat R package. Gene expression data were normalized, highly variable genes were identified, and principal component analysis (PCA) was conducted. The first 15 principal components were used for unsupervised clustering, and Uniform Manifold Approximation and Projection (UMAP) was applied for dimensionality reduction and visualization of major immune cell populations.

### Cell type annotation and compositional analysis

Cell clusters were annotated based on the expression of canonical marker genes, enabling reliable identification of major immune cell populations, including T cells, B cells, NK cells, CD14^+^ monocytes, CD16^+^ monocytes, and neutrophils. *ELF4*-mutant-assigned and wild-type-assigned cells were overlaid onto the UMAP representation to assess their distribution across immune compartments. The distributions of mutant-assigned, wild-type-assigned, and uncovered cells across annotated immune-cell subsets were summarized descriptively.

### Differential gene expression analysis

Differential gene expression analyses were performed within each immune cell subset by comparing *ELF4*-mutant-assigned cells with wild-type-assigned cells from the same patient using the FindMarkers function in Seurat. Genes with a P value < 0.05 and an absolute log2 fold change > 0.1 were considered differentially expressed. Heatmaps were generated using the ggplot2 and heatmap.2 packages in R.

### Single-cell mutation identification and analysis

Single-cell mutation analysis was performed on aligned sequencing data generated from the single-cell RNA sequencing pipeline. Single-nucleotide variants were identified at the single-cell level using the Pysam-based tool cb_sniffer with default parameters. Reads lacking valid cell barcode (CB) or unique molecular identifier (UMI; UB) tags were excluded (28, 29). For each UMI at a given genomic position, base calls were aggregated, and a mutation was assigned when a single base accounted for at least 75% of supporting reads. UMIs not meeting this criterion were excluded from downstream analyses.

At the *ELF4* c.239T>A position, cells were classified as mutant-assigned or wild-type-assigned according to the informative UMI-level allele calls. Cells without sequencing reads covering the variant position were classified as uncovered. Uncovered cells were excluded from allele-assignment-based differential-expression and pathway-enrichment analyses (27). Because the assignments were based on expressed RNA and were not validated by matched single-cell DNA sequencing, they represent transcript-based allele assignments rather than definitive genomic genotypes.

### Pathway enrichment analyses

To interpret the biological significance of transcriptional differences between *ELF4*-mutant-assigned and wild-type-assigned cells, Gene Ontology (GO) biological process and Kyoto Encyclopedia of Genes and Genomes (KEGG) pathway enrichment analyses were performed on differentially expressed genes within each immune cell subset using the clusterProfiler R package. Pathways and GO terms with adjusted P values < 0.05 were considered significantly enriched.

### Statistical analysis

Comparisons among multiple groups were performed using ordinary one-way ANOVA followed by Tukey’s multiple-comparisons test in GraphPad Prism 10.00. Error bars represent SD. P value < 0.05 was considered statistically significant.

## Results

### Clinical manifestations of the patient

The proband (II.1) is an 8-year-old boy who developed recurrent painful oral ulcers at approximately 3 years of age. Recurrent febrile episodes began at approximately 6 years of age, each lasting 1–3 days and recurring at intervals of approximately 2 weeks. No genital, perianal, or ocular ulcers were observed during the disease course. Growth and development were appropriate for age; at the most recent evaluation, his height was 134.3 cm and weight was 32 kg. Physical examination revealed several ulcers involving the lips and tongue, without genital or perianal ulceration or joint swelling.

Laboratory investigations performed during an active febrile episode revealed an increased C-reactive protein level of 20.02 mg/L (normal range: 0–10 mg/L). Complete blood count was unremarkable, with a white blood cell count of 5.23 × 10^9^/L, hemoglobin of 130 g/L, and platelet count of 224 × 10^9^/L. Antinuclear and anti-double-stranded DNA antibodies were negative. Lymphocyte subset analysis and serum immunoglobulin levels were within the corresponding reference ranges (**Supplementary Table 3**). Treatment with thalidomide (25 mg once daily) resulted in good control of recurrent fever and oral ulcers, with normalization of inflammatory markers during follow-up.

### Identification of a truncating *ELF4* p.L80* variant

**Figure 1:**
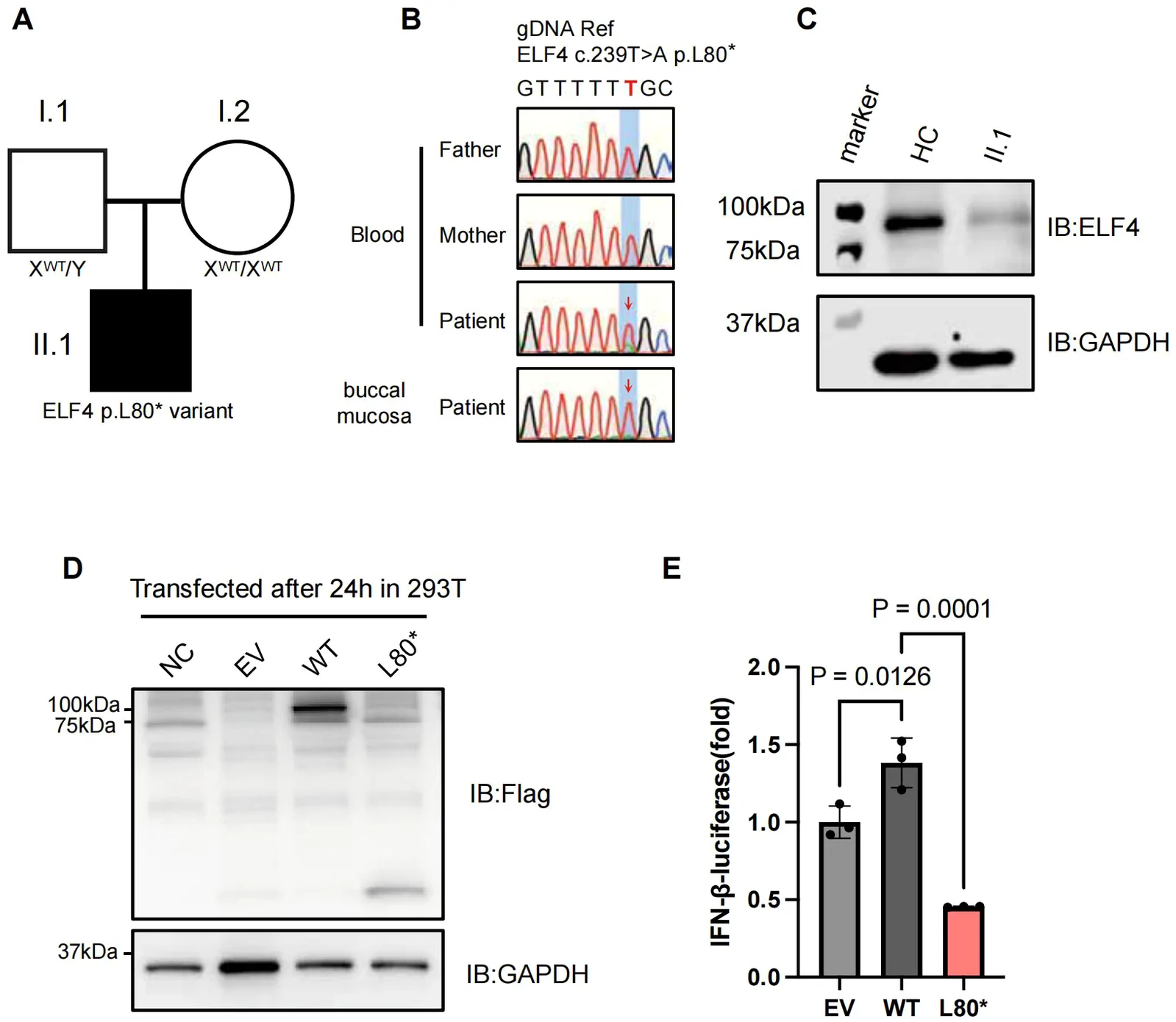
Identification and functional characterization of an *ELF4* p.L80* variant. **(A)** Pedigree of the family showing the affected male proband (II.1) harboring the *ELF4* p.L80* variant, while both parents are unaffected. **(B)** Sanger sequencing chromatograms showing the *ELF4* c.239T>A (p.L80*) variant detected in peripheral blood cells of the patient but not detected in parental peripheral blood samples or the patient’s buccal mucosa by Sanger sequencing. **(C)** Immunoblot analysis of endogenous ELF4 protein expression in PBMCs from a healthy control (HC) and the patient (II.1). **(D)** Immunoblot analysis of FLAG-tagged wild-type (WT) and p.L80*-mutant *ELF4* (L80*) expressed in HEK293T cells (NC, negative control; EV, empty vector). **(E)** IFN-β promoter dual-luciferase reporter assay evaluating the transcriptional activity of the *ELF4* p.L80* variant. Luciferase activity was measured 24 h after transfection, and firefly luciferase activity was normalized to Renilla luciferase activity. Data were pooled from three independent experiments and are presented as mean ± SD.

Given the patient’s recurrent unexplained inflammatory manifestations, whole-exome sequencing was performed to investigate a potential genetic etiology. Genetic analysis identified a truncating variant in *ELF4* (c.239T>A, p.L80*) in peripheral blood cells from individual II.1 (**Figure 1A**), which was not detected in parental peripheral blood samples. The variant was also not detected in the proband’s buccal mucosa by Sanger sequencing (**Figure 1B**). Although this tissue-discordant detection pattern raises the possibility of mosaicism, conventional Sanger sequencing has limited sensitivity for detecting low-level variants. Therefore, the developmental origin and tissue distribution of the *ELF4* p.L80* variant cannot be definitively established from the available samples. The variant introduces a premature stop codon in the N-terminal region of ELF4, predicting a severely truncated protein lacking critical functional domains.

### *ELF4* p.L80* variant shows loss-of-function effects

To evaluate the functional impact of the *ELF4* p.L80* variant, we first examined endogenous ELF4 expression. Immunoblot analysis showed a reduced full-length ELF4 signal in patient-derived PBMCs compared with the healthy control (**Figure 1C**). We next transiently expressed FLAG-tagged wild-type or p.L80* mutant ELF4 in HEK293T cells. Immunoblot analysis showed a markedly reduced full-length ELF4 signal in cells expressing the p.L80* construct compared with wild-type ELF4 (**Figure 1D**).

To assess the functional impact of the p.L80* variant, we performed an IFN-β promoter luciferase reporter assay. Wild-type ELF4 significantly enhanced reporter activity compared with the empty-vector control, whereas p.L80* showed markedly reduced activity relative to wild-type ELF4 (**Figure 1E**), supporting impaired ELF4-mediated transcriptional activation.

Together, the reduced full-length ELF4 signal observed in patient-derived PBMCs and in the p.L80* overexpression system, together with impaired IFN-β promoter activation, support a loss-of-function effect of the *ELF4* p.L80* variant, consistent with previous studies (6, 7). In contrast to germline ELF4 deficiency (3, 6–13), this patient exhibited a relatively restricted clinical phenotype. However, given the unresolved developmental origin and tissue distribution of the p.L80* variant, this phenotypic difference cannot be attributed to mosaicism and should be interpreted descriptively. Other factors, including mutation position, residual ELF4 function, genetic modifiers, and interindividual variability, may also contribute to disease severity. This descriptive comparison is summarized in **Table 1**.

**Table 1:** Descriptive comparison of clinical characteristics between individual II.1 and previously reported patients with germline ELF4 deficiency (DEX).

Clinical feature	Individual II.1 (this study)	Reported DEX patients (germline ELF4 LOF)
Sex	Male	Predominantly male
Genetic status	*ELF4* truncating variant (c.239T>A, p.L80*) detected in peripheral blood	Germline ELF4 loss-of-function variants
Mode of inheritance	Undetermined; variant not detected in parental blood samples	X-linked (inherited or de novo)
Age at onset	3 years old	Early childhood to adolescence
Oral ulcers	Present, recurrent	Common
Genital / perianal ulcers	Absent	Variable
Ocular involvement	Absent	Rare
Recurrent fever	Present	Common
Gastrointestinal involvement	Absent	Common (IBD-like colitis)
Skin manifestations	Absent	Variable
Arthritis / arthralgia	Absent	Variable
Inflammatory markers	Elevated CRP	Elevated CRP and/or ESR
Autoantibodies	ANA and anti-dsDNA negative	Typically negative
Lymphocyte subsets	Normal	Variable
Humoral immunity	Normal	Variable
Treatment	Thalidomide (effective)	Steroids, biologics (anti–IL-1, anti–TNF, anti–IL-12/23), others
Clinical course	Well controlled	Chronic, relapsing

### Full-length single-cell transcriptomic analysis reveals transcriptional alterations in *ELF4* allele-assigned immune cells

**Figure 2:**
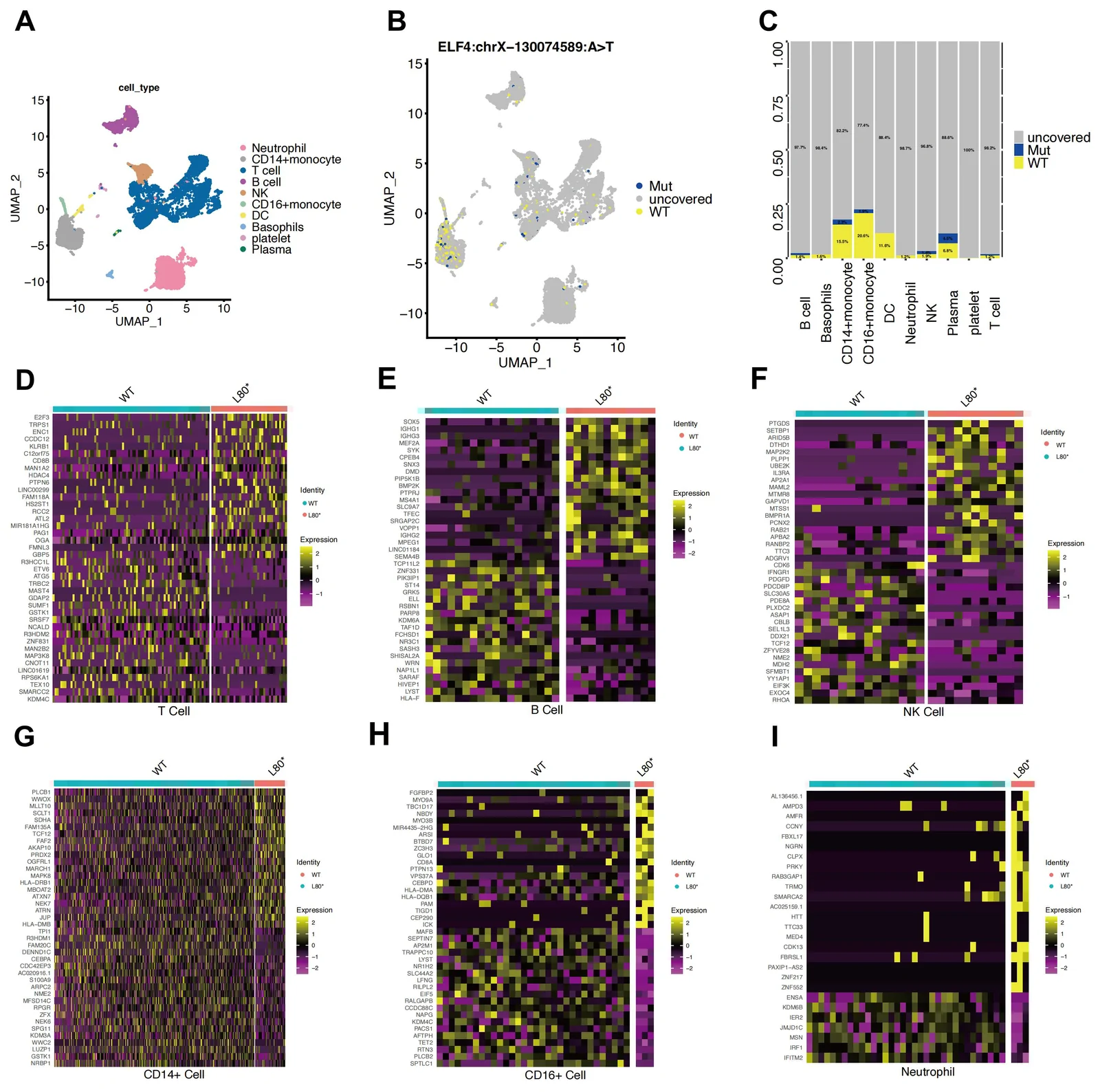
Single-cell analysis of transcript-based *ELF4* allele assignments across peripheral blood immune populations. **(A)** UMAP visualization of single-cell RNA-sequencing data from patient whole-blood leukocytes, colored by major immune cell types. **(B)** UMAP projection showing the distribution of *ELF4* p.L80* mutant-assigned, wild-type-assigned, and uncovered cells. **(C)** Distribution of allele-assignment categories within each immune population. Exact cell numbers are provided in **Supplementary Table 1**. **(D–F)** Heatmaps showing differentially expressed genes between mutant-assigned and wild-type-assigned cells in T cells, B cells, and NK cells. **(G–I)** Heatmaps showing differentially expressed genes between mutant-assigned and wild-type-assigned cells in CD14^+^ monocytes, CD16^+^ monocytes, and neutrophils. Because only three mutant-assigned cells were available in both the CD16^+^ monocyte and neutrophil subsets, analyses of these subsets are exploratory and should be interpreted cautiously.

To characterize the transcriptional landscape associated with the *ELF4* p.L80* variant at single-cell resolution, we performed full-length single-cell RNA sequencing on whole-blood leukocytes from individual II.1. Unsupervised clustering identified the major immune populations, including T cells, B cells, NK cells, CD14^+^ monocytes, CD16^+^ monocytes, neutrophils, dendritic cells, basophils, plasma cells, and platelets (**Figure 2A**).

Projection of mutant-assigned, wild-type-assigned and uncovered cells onto the UMAP representation showed that mutant-assigned cells were distributed across multiple immune lineages, with variable representation among cell subsets (**Figures 2B, C**). Because sequencing coverage at the *ELF4* c.239T>A locus varied among individual cells, the exact numbers of mutant-assigned, wild-type-assigned, and uncovered cells in each annotated subset are provided in **Supplementary Table 1**. Locus-specific sequencing depth and mutant- and wild-type-supporting reads and UMIs are summarized in **Supplementary Table 2**.

We next performed differential gene expression analysis within each immune subset. Transcriptional differences were observed in lymphoid populations, including T, B, and NK cells, as well as in myeloid populations, including CD14^+^ monocytes, CD16^+^ monocytes, and neutrophils (**Figures 2D–I**). However, the numbers of allele-informative cells varied substantially among subsets. In particular, limited CD16^+^ monocytes and neutrophils were classified as mutant-assigned; therefore, the differential-expression results from these subsets should be interpreted as preliminary.

### ELF4-regulated genes and cell type-specific transcriptional programs associated with the *ELF4* p.L80* variant

**Figure 3:**
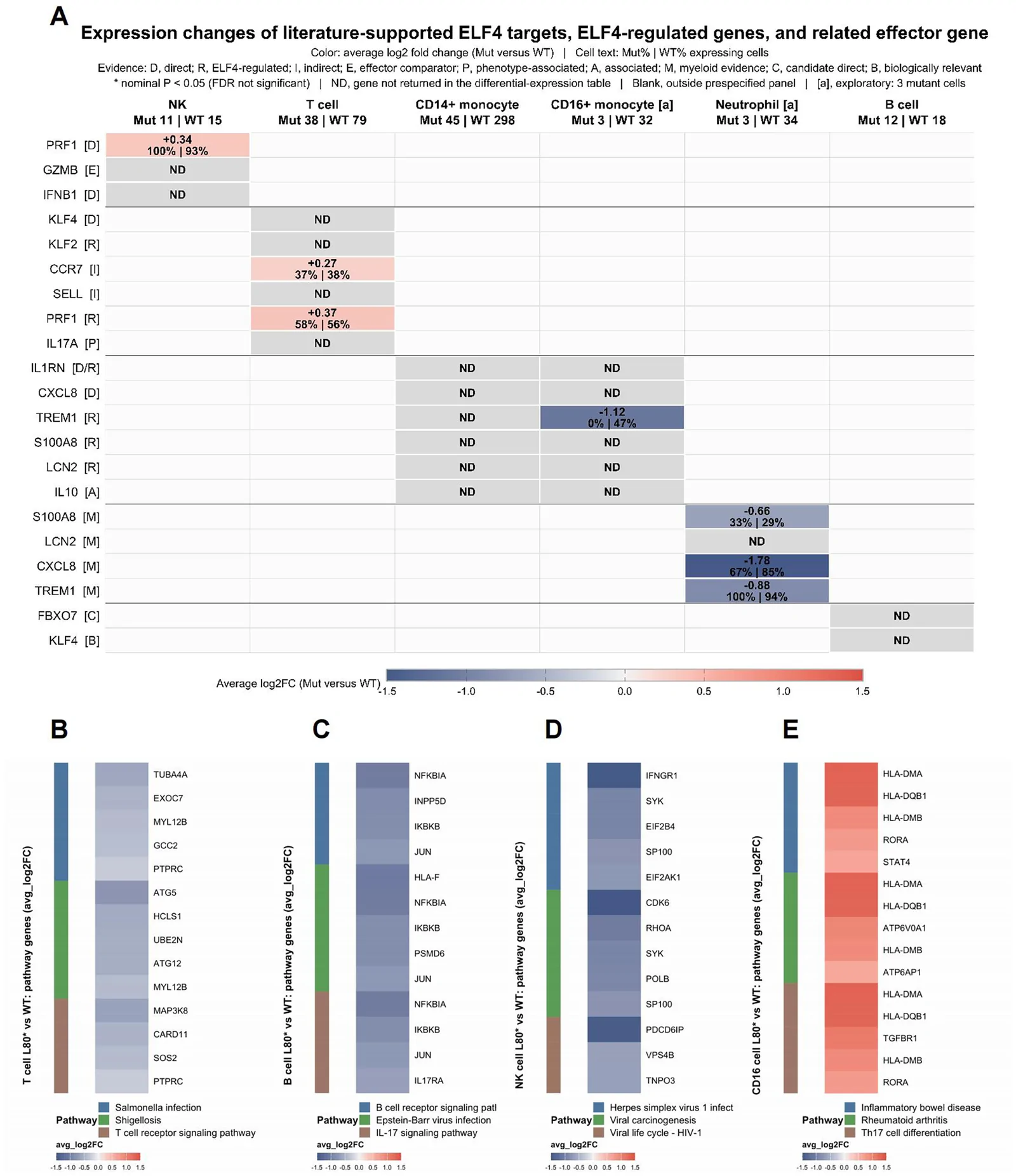
Analysis of ELF4-regulated genes and pathway-associated transcriptional changes in ELF4 allele-assigned immune subsets. **(A)** Expression changes of literature-supported ELF4 targets, ELF4-regulated genes, and related effector genes in mutant-assigned versus wild-type-assigned immune subsets. Color indicates average log2 fold change, and cell text shows the percentages of mutant-assigned and wild-type-assigned cells expressing each gene. Evidence categories are indicated as direct target (D), ELF4-regulated (R), indirect (I), effector comparator (E), phenotype-associated (P), ELF4-associated (A), myeloid-supported (M), candidate direct target (C), or biologically relevant (B). ND indicates that the gene was not returned in the corresponding differential-expression table. **(B–E)** Heatmaps of representative pathway-associated genes in T cells **(B)**, B cells **(C)**, NK cells **(D)**, and CD16^+^ monocytes **(E)**. Colors represent average log2 fold changes. Analyses of CD16^+^ monocytes and neutrophils are exploratory because only three mutant-assigned cells were available in each subset.

To further relate the observed transcriptional alterations to established ELF4 biology, we performed analysis of selected ELF4 transcriptional targets, ELF4-regulated genes, and functionally related genes across immune-cell subsets (1–5, 30–33) (**Figure 3A**). Several genes showed cell type-specific expression changes. *PRF1* was modestly increased in mutant-assigned NK and T cells, whereas *TREM1* was reduced in mutant-assigned CD16^+^ monocytes. In neutrophils, *S100A8*, *CXCL8*, and *TREM1* also showed reduced expression. In contrast, several established ELF4-regulated genes, including *IFNB1, IL1RN, KLF4*, and *KLF2*, were not returned in the corresponding differential-expression analyses. Thus, a uniform canonical ELF4 target-gene signature was not observed across immune-cell subsets. Because the present analysis reflects steady-state transcript abundance in circulating immune cells, the absence of uniform downregulation does not necessarily indicate preserved ELF4 transcriptional activity.

We next examined representative genes underlying the major cell type-specific pathway alterations (**Figures 3B–E**). In T and B cells, transcriptional changes were mainly related to lymphocyte receptor signaling and immune activation (**Figures 3B, C**).

In mutant-assigned NK cells, genes associated with antiviral and interferon-related programs were downregulated, including reduced expression of the interferon-response gene *IFNGR1*, suggesting impaired antiviral transcriptional programs (**Figure 3D**). In contrast, exploratory analysis of mutant-assigned CD16^+^ monocytes showed increased expression of genes contributing to Th17-cell differentiation and inflammatory bowel disease-related pathways, suggesting enhanced inflammation-related transcriptional programs in this subset (**Figure 3E**). However, because only three CD16^+^ monocytes were classified as mutant-assigned, these findings should be considered exploratory and hypothesis-generating rather than evidence of a defined pathogenic mechanism. Validation in additional patients and larger numbers of allele-informative cells will be required.

Broader GO and KEGG enrichment analyses are provided in **Supplementary Figures 1** and **2**. To further characterize inflammation-related transcriptional changes across immune subsets, we additionally examined genes involved in NF-κB and Toll-like receptor signaling, inflammatory responses, and IBD/Th17/Th1/Th2-related pathways (**Supplementary Figure 3**). These genes showed heterogeneous, cell type-specific alterations rather than uniform activation or suppression of a single inflammatory pathway, further supporting cell type-specific transcriptional effects associated with the *ELF4* p.L80* variant.

## Discussion

In this study, we describe a pediatric patient with a truncating *ELF4* p.L80* variant detected in peripheral blood, recurrent mucocutaneous inflammation, and periodic fever. Germline loss-of-function mutations in *ELF4* are known to cause DEX, a disorder characterized by heterogeneous inflammatory manifestations involving mucosal, gastrointestinal, and systemic features. Compared with previously reported germline cases (1, 3, 6–13), the present patient exhibited a relatively restricted clinical phenotype. Given that the developmental origin and tissue distribution of the p.L80* variant remain unresolved, this phenotypic difference cannot be attributed to mosaicism. Nevertheless, the tissue-discordant detection pattern leaves open the possibility of a somatic mosaic origin, which will require more sensitive DNA-based analysis for confirmation. Other factors, including mutation position, residual ELF4 function, genetic modifiers, and interindividual variability, may also contribute to phenotypic severity. Notably, our functional analyses support a loss-of-function effect of the *ELF4* p.L80* variant, although the extent to which residual ELF4 function contributes to the clinical phenotype remains uncertain.

Establishing a genetic diagnosis of ELF4 deficiency may also have implications for clinical management. Recognition of DEX as a monogenic autoinflammatory disorder can facilitate appropriate longitudinal surveillance for inflammatory and immune manifestations and may help guide individualized treatment decisions. Previous patients with DEX have shown variable responses to conventional anti-inflammatory therapies and biologic agents targeting TNF-α, IL-1, or IL-12/23 pathways (3, 6–11). In the present patient, recurrent fever and oral ulcers were well controlled with thalidomide. However, no ELF4-specific treatment strategy has yet been established, and therapeutic selection should therefore remain guided by the individual clinical phenotype and disease severity.

By applying full-length single-cell RNA sequencing, we compared mutant-assigned and wild-type-assigned immune cells from the same individual, thereby assessing mutation-associated transcriptional programs while minimizing interindividual variability. Distinct transcriptional profiles were observed across both lymphoid and myeloid compartments, supporting cell type-specific transcriptional alterations associated with the *ELF4* p.L80* variant.

However, analysis of ELF4 targets and ELF4-regulated genes did not reveal a uniform canonical target-gene signature across immune subsets. Several established ELF4-regulated genes were not identified as differentially expressed, whereas PRF1 was not reduced in mutant-assigned NK or T cells. This lack of uniform downregulation does not necessarily contradict the established transcriptional activity of ELF4, because our single-cell analysis reflects steady-state transcript abundance in circulating immune cells rather than promoter activity, protein expression, or stimulus-induced transcription. Expression of ELF4-regulated genes is likely to be highly cell type- and context-dependent and may also be influenced by secondary or compensatory inflammatory and interferon-response pathways. Thus, the observed transcriptomic patterns may reflect a combination of direct ELF4-dependent effects and secondary transcriptional responses rather than a uniform loss of canonical target-gene expression.

At the pathway level, the transcriptional effects associated with the *ELF4* p.L80* variant were also cell type specific rather than uniform across the immune system. In adaptive immune cells such as T cells and B cells, altered pathways were primarily related to immune activation and lymphocyte signaling. Innate immune subsets also showed distinct transcriptional alterations. Previous studies in patients with DEX and ELF4-deficient experimental models have shown that ELF4 is required for NK-cell development, terminal maturation, and cytotoxic function (2, 30). In the myeloid compartment, ELF4 deficiency impairs anti-inflammatory transcriptional programs and enhances inflammatory responses in macrophages (3). Consistent with these established functions, *ELF4* p.L80*-mutant-assigned NK cells in our patient showed downregulation of antiviral and interferon-related pathways, together with reduced IFNGR1 expression. Conversely, exploratory analysis of mutant-assigned CD16^+^ monocytes suggested enrichment of pro-inflammatory pathways associated with Th17-cell differentiation and inflammatory bowel disease.

Collectively, these findings suggest divergent cell type-specific transcriptional biases observed in *ELF4* p.L80*-assigned cells, characterized by attenuated antiviral and interferon-related programs in NK cells (1, 2) and preliminary inflammation-related transcriptional changes in CD16^+^ monocytes. By extending observations from germline DEX and ELF4-deficient experimental models to *ELF4* p.L80*-assigned immune cells within the same individual, our study further supports a context-dependent role of ELF4 in innate immune homeostasis. However, the CD16^+^ monocyte analysis was based on a limited number of mutant-assigned cells, and these findings should therefore be considered hypothesis-generating rather than definitive evidence of a pathogenic mechanism. Rather than implicating a single pathogenic mechanism, these findings suggest that the *ELF4* p.L80* variant is associated with differential transcriptional programs across immune-cell subsets.

However, the developmental origin and tissue distribution of the *ELF4* p.L80* variant remain unresolved. Although the variant was detected in peripheral blood and was not detected in buccal mucosa by Sanger sequencing, the limited sensitivity of this method does not exclude low-level variant representation in buccal mucosa or other tissues. Moreover, the single-cell RNA-sequencing analyses provide transcript-based allele assignments rather than genomic measurements of variant allele frequency. Because sensitive DNA-based quantification in additional tissues and purified immune-cell subsets was not available, we cannot definitively determine whether the variant is constitutional or represents somatic mosaicism, nor define its lineage distribution. Future studies using ddPCR or deep sequencing in multiple tissues and defined immune populations will be required to resolve the developmental origin and tissue distribution of the *ELF4* p.L80* variant.

## Data Availability

The data sets for this article are not publicly available due to concerns regarding participant/patient anonymity and genetic privacy. Requests to access the datasets should be directed to the corresponding authors, Hongqiang Du (doctordo@aliyun.com) and Xiaodong Zhao (zhaoxd530@aliyun.com), and will be considered subject to appropriate ethical and privacy requirements.
