# Supplementary material for: A truncating ELF4 variant in a pediatric patient with autoinflammatory disease and cell type-specific immune alterations
Source: Front Immunol. 2026 Sep 7;17:1859084. doi: 10.3389/fimmu.2026.1859084 (PMC13590361; doi:10.3389/fimmu.2026.1859084)
Supplement: Supplementary file 1 [file SupplementaryFile1.docx]

**A truncating *ELF4* variant in a pediatric patient with autoinflammatory disease and cell type-specific immune alterations Supplementary Data**

**Figure S1. Gene Ontology (GO) and KEGG pathway enrichment analyses of downregulated differentially expressed genes in *ELF4* mutant-assigned immune cell populations.** Downregulated genes were identified by comparing mutant-assigned and wild-type-assigned cells within each immune cell subset from single-cell RNA sequencing data and subjected to Gene Ontology (GO) biological process and Kyoto Encyclopedia of Genes and Genomes (KEGG) pathway enrichment analyses. GO and KEGG analyses revealed that downregulated genes in T cells, B cells, NK cells, CD14⁺ monocytes, and CD16⁺ monocytes were predominantly enriched in pathways related to immune regulation, antiviral and interferon responses, transcriptional and translational control, chromatin organization, autophagy, cytoskeletal organization, and hematopoietic or leukocyte differentiation. In KEGG dot plots, dot size represents the number of enriched genes, and color indicates the adjusted P value, while GO bar plots display significantly enriched biological processes within each cell type. Collectively, these analyses show cell type-specific enrichment of downregulated genes across immune and stress-response pathways.

**Figure S2. Gene Ontology (GO) and KEGG pathway enrichment analyses of upregulated differentially expressed genes in *ELF4* mutant-assigned immune cell populations.** Upregulated genes were identified by comparing mutant-assigned and wild-type-assigned cells within each immune cell subset from single-cell RNA sequencing data and subjected to Gene Ontology (GO) biological process and Kyoto Encyclopedia of Genes and Genomes (KEGG) pathway enrichment analyses. GO and KEGG analyses revealed that upregulated genes in T cells, B cells, NK cells, CD14⁺ monocytes, CD16⁺ monocytes, and neutrophils were predominantly enriched in pathways related to cell cycle regulation, antigen processing and presentation, immune receptor signaling, autophagy and mitophagy, RNA metabolism, mitochondrial function, vesicle-mediated transport, and inflammatory or stress-response pathways. In KEGG dot plots, dot size represents the number of enriched genes, and color indicates the adjusted P value, while GO bar plots display significantly enriched biological processes within each immune cell subset. Collectively, these analyses show cell type-specific enrichment of upregulated genes across immune, metabolic, and stress-response pathways.

**Figure S3. Cell type-specific alterations in inflammation-related transcriptional programs in *ELF4* allele-assigned immune cells.**

**(A)** Expression changes of genes involved in canonical and non-canonical NF-κB signaling across immune-cell subsets. **(B)** Expression changes of Toll-like receptor signaling-associated genes. **(C)** Expression changes of selected inflammatory-response genes, including chemokine-, AP-1-, JAK–STAT-, interferon-, and myeloid inflammation-related genes. **(D)** Expression changes of representative genes associated with inflammatory bowel disease and Th17/Th1/Th2 differentiation pathways. Colors indicate average log2 fold changes in mutant-assigned versus wild-type-assigned cells, and values within cells indicate the percentages of mutant-assigned and wild-type-assigned cells expressing each gene. ND indicates that the gene was not returned in the corresponding differential-expression analysis. Asterisks indicate nominal P < 0.05 without significance after FDR correction. Analyses of CD16⁺ monocytes and neutrophils are exploratory because only three mutant-assigned cells were available in each subset.

| **Cell subset** | **Mutant** | **WT** | **Uncovered** | **Total** |
| --- | --- | --- | --- | --- |
| Neutrophils | 3 | 34 | 2785 | 2822 |
| CD14**^+^** monocytes | 45 | 298 | 1584 | 1927 |
| T cells | 38 | 79 | 6252 | 6369 |
| B cells | 12 | 18 | 1280 | 1310 |
| NK cells | 11 | 15 | 777 | 803 |
| CD16**^+^** monocytes | 3 | 32 | 120 | 155 |
| Dendritic cells | 0 | 17 | 130 | 147 |
| Basophils | 0 | 2 | 123 | 125 |
| Plasma cells | 2 | 3 | 39 | 44 |
| Platelets | 0 | 0 | 85 | 85 |

**Table S1. Distribution of transcript-based *ELF4* p.L80* allele assignments across peripheral blood immune cell subsets.** Cells were classified as mutant-assigned, wild-type (WT)-assigned or uncovered according to the single-cell RNA-sequencing reads spanning the *ELF4* c.239T>A variant site. “Uncovered” indicates cells without sufficient sequencing coverage at the variant position for reliable allele assignment. Total represents the total number of cells identified within each annotated cell subset. These RNA-based assignments should not be interpreted as definitive genomic genotypes or genomic variant allele frequencies.

| **Cell subset** | **Mean depth** | **Median depth (IQR)** | **Mutant-supporting reads** | **WT-supporting reads** | **Mutant-supporting UMIs** | **WT-supporting UMIs** |
| --- | --- | --- | --- | --- | --- | --- |
| Neutrophils | 1.83 | 1.0  (1-2) | 14 | 74 | 7 | 42 |
| CD14**^+^** monocytes | 2.51 | 2.0  (1-3) | 147 | 790 | 72 | 378 |
| T cells | 2.59 | 2.0 (1-3) | 107 | 237 | 45 | 94 |
| B cells | 2.66 | 2.0 (1-3) | 25 | 60 | 12 | 20 |
| NK cells | 2.03 | 2.0 (1-3) | 23 | 36 | 12 | 17 |
| CD16**^+^** monocytes | 2.26 | 1.0 (1-3) | 6 | 71 | 3 | 39 |
| Basophils | 1.50 | 1.5  (1-1) | 0 | 3 | 0 | 2 |
| Plasma cells | 3.00 | 1.0 (1-1) | 2 | 16 | 2 | 4 |

**Table S2. Sequencing depth and allele-supporting reads and UMIs at the *ELF4* c.239T>A locus across immune-cell subsets**. Sequencing depth and mutant- or wild-type-supporting reads and unique molecular identifiers (UMIs) were quantified from aligned single-cell RNA-sequencing BAM files. Supporting UMI counts represent transcript-level molecular evidence and are not equivalent to allele-assigned cell numbers. These RNA-based measurements provide technical support for *ELF4* allele assignment but should not be interpreted as genomic variant allele frequencies.

| **Category** | **Parameter** | **Result** | **Reference value** | **Unit** |
| --- | --- | --- | --- | --- |
| Lymphocyte subsets | CD3+% | 70.5 | 55-84 | % |
|  | CD3+CD4+% | 30.3 | 31-60 | % |
|  | CD3+CD8+% | 36 | 13-41 | % |
|  | CD3+CD4+CD8+% | 0.3 | 0-2 | % |
|  | CD3+CD4+/CD3+CD8+ | 0.84 | 0.68-2.47 | - |
|  | CD19+% | 15.1 | 6-25 | % |
|  | CD56+% | 12.8 | 5-27 | % |
| Absolute lymphocyte counts | Total lymphocyte count | 2265 | 1530-3700 | cells/μL |
|  | CD3+# | 1597 | 770-2860 | cells/μL |
|  | CD4+# | 686 | 414-1440 | cells/μL |
|  | CD8+# | 815 | 238-1250 | cells/μL |
|  | CD4+CD8+# | 7 | 0-30 | cells/μL |
|  | CD19+# | 342 | 90-560 | cells/μL |
|  | CD56+# | 289 | 150-1110 | cells/μL |
| Serum  immunoglobulins | IgG | 11.17 | 6.7-15.3 | g/L |
|  | IgA | 0.84 | 0.52-2.74 | g/L |
|  | IgM | 0.88 | 0.48-2.31 | g/L |
|  | IgE | 10.52 | 0-90 | IU/ml |

**Table S3. Immunological evaluation of the patient.** Lymphocyte subset proportions and absolute counts, together with serum immunoglobulin levels are presented with the corresponding laboratory reference ranges.
